# Dutch senior medical students and disaster medicine: a national survey

**DOI:** 10.1186/s12245-015-0077-0

**Published:** 2015-09-03

**Authors:** Luc J. M. Mortelmans, Stef J. M. Bouman, Menno I. Gaakeer, Greet Dieltiens, Kurt Anseeuw, Marc B. Sabbe

**Affiliations:** Department of Emergency Medicine ZNA camp Stuivenberg, Lange Beeldekensstraat 267, B2060 Antwerp, Belgium; Center for Research and Education in Emergency Care, Leuven, Belgium; Department of Emergency Medicine Admiraal De Ruyterhospital, Goes, The Netherlands; Department of Emergency Medicine University Hospital Gasthuisberg, Leuven, Belgium

**Keywords:** Education, Disaster medicine, Medical students, Curriculum

## Abstract

**Background:**

Medical students have been deployed in victim care of several disasters throughout history. They are corner stones in first-line care in recent pandemic planning. Furthermore, every physician and senior medical student is expected to assist in case of disaster situations, but are they educated to do so? Being one of Europe’s densest populated countries with multiple nuclear installations, a large petrochemical industry and also at risk for terrorist attacks, The Netherlands bear some risks for incidents. We evaluated the knowledge on Disaster Medicine in the Dutch medical curriculum. Our hypothesis is that Dutch senior medical students are not prepared at all.

**Methods:**

Senior Dutch medical students were invited through their faculty to complete an online survey on Disaster Medicine, training and knowledge. This reported knowledge was tested by a mixed set of 10 theoretical and practical questions.

**Results:**

With a mean age of 25.5 years and 60 % females, 999 participants completed the survey. Of the participants, 51 % considered that Disaster Medicine should absolutely be taught in the regular medical curriculum and only 2 % felt it as useless; 13 % stated to have some knowledge on disaster medicine. Self-estimated capability to deal with various disaster situations varied from 1.47/10 in nuclear incidents to 3.92/10 in influenza pandemics. Self-estimated knowledge on these incidents is in the same line (1.71/10 for nuclear incidents and 4.27/10 in pandemics). Despite this limited knowledge and confidence, there is a high willingness to respond (ranging from 4.31/10 in Ebola outbreak over 5.21/10 in nuclear incidents to 7.54/10 in pandemics). The case/theoretical mix gave a mean score of 3.71/10 and raised some food for thought. Although a positive attitude, 48 % will place contaminated walking wounded in a waiting room and 53 % would use iodine tablets as first step in nuclear decontamination. Of the participants, 52 % even believes that these tablets protect against external radiation, 41 % thinks that these tablets limit radiation effects more than shielding and 57 % believes that decontamination of chemical victims consists of a specific antidote spray in military cabins.

**Conclusions:**

Despite a high willingness to respond, our students are not educated for disaster situations.

**Electronic supplementary material:**

The online version of this article (doi:10.1186/s12245-015-0077-0) contains supplementary material, which is available to authorized users.

## Background

In the past, medical students have been involved in direct patient care in large-scale mass casualty incidents. From the Spanish flu pandemic in 1918 [1] over floodings [2], devastating earthquakes [3, 4] to the 9/11 massacre [5], medical students have been deployed in victim care. The Belgian Royal Academy of Medicine even mentioned them as an important player in the national H5N1 pandemic plan in 2005 [6] although they were absolutely not prepared for it [7]. Despite the expectation of voluntary deployment, we know that training in Disaster Medicine has little to no place in regular medical curricula worldwide [8–16]. How can we rely on their help if they are not prepared? Our hypothesis is that, in the Netherlands, senior medical students are minimally prepared for direct patient care or other tasks during mass casualty incidents.

## Methods

To evaluate Disaster Medicine education amongst senior medical students, a descriptive cross-sectional study was performed in the academic year 2013–2014. The study was approved by the local ethical committee of ZNA, Antwerp.

Senior medical students (last 2 years of the 6 years of medical education) of the eight medical faculties that provide full medical training in The Netherlands were invited through their faculty and/or social media to complete an online survey (Survey Monkey, Palo Alto, California USA) on Disaster Medicine, training and knowledge. The survey (see Additional file 1: Figure S1) consisted of demographic data, prior education and self-estimated knowledge on and capability to deal with several disaster scenarios as well as their willingness to work in these circumstances. Scores were given on a scale from 0 to 10. This reported knowledge was tested by a mixed set of 10 theoretical questions and practical cases, each correct answer valuing 1 point out of 10. The survey was developed at the Center for Research and Education in Emergency Care (CREEC) at the University of Leuven based upon literature data and validated by several disaster specialists from the network of the CREEC and the Leuven University Disaster Management Course (joint venture with the Belgian Military and the Flemish Society of Emergency Nurses).

The data were statistically evaluated by the use of Stata SE 10.1 (StataCorp LP, College Station, Texas USA). We used where appropriate, the Pearson chi-square test, the two-sided *t* test, the Wilcoxon–Mann–Whitney test, the Kruskal–Wallis test and the Pearson and Spearman correlation coefficients. A *p* value smaller than 0.05 was considered to be significant.

Local student organisations were contacted to check to which extent Disaster Medicine courses (obligatory or voluntary) were incorporated in the local curriculum.

## Results

Unfortunately, we could only approach students from six out of eight faculties as we were not allowed to contact students from both faculties in Amsterdam due to so-called survey overload. On a total population of 4408, 999 students participated, being a response rate of 22.66 %. Demographic data are grouped in Table 1. Self-estimated knowledge on and capability to deal with some specific disaster situations as well as willingness to assist in these situations during their apprenticeship are listed in Table 2. The mean score on the theoretical/case mix was 3.71/10 (0–10 SD 2.56), an overview of the questions and all results is given in Table 3. Some topics here are certainly food for thought; 48 % directs potentially contaminated patients into the waiting room with all other patients at risk for contamination. There is a huge belief in the effects of iodine tablets: 52 % is convinced that they protect against external radiation and up to 53 % will use them as a first step in nuclear decontamination. Where 54 % knows that that limiting time of exposure, increasing distance and shielding limits radiation damage the most, up to 41 % will use iodine tablets for this purpose; 57 % believes that decontamination of chemical victims consists of a specific antidote spray in military cabins.

**Table 1:** Demographic data of our study population

Gender	Male	41 %
	Female	59 %
Mean age		25.54 (20–49)
Study year	5th	50 %
	6th (last)	50 %
Future orientation	Family practice	38 %
	Occupational/insurance	2 %
	Specialisation	60 %
Lives within 20 km of nuclear installation	Yes	2 %
	No	69 %
	Don’t know	29 %
Lives within 20 km of chemical installation	Yes	16 %
	No	28 %
	Don’t know	56 %
Any EMS/DM experience	Yes	7 %
	No	93 %
Has some DM knowledge	Yes	13 %
	No	87 %
DM needs to be trained within curriculum	Absolutely	51 %
	Useful	48 %
	Useless	1 %

**Table 2:** Scores in mean (minimum–maximum) of the 0–10 visual analogical scale on self-estimated knowledge and capabilty and willingness to respond in the evaluated disaster situations

	Self-estimated knowledge	Self-estimated capability	Willingness to respond
Nuclear incidents	1.71/10 (0–8)	1.47/10 (0–9)	5.21/10 (0–10)
Chemical incidents	2.28/10 (0–8)	1.85/10 (0–8)	5.87/10 (0–10)
Biological incidents	2.28/10 (0–8)	2.04/10 (0–8)	6.61/10 (0–10)
Outbreak very infectious disease (e.g. N5H1)	4.27/10 (0–10)	3.92/10 (0–9)	7.54/10 (0–10)
Outbreak very dangerous contagious infection (e.g. Ebola)	2.88/10 (0–10)	2.47/10 (0–9)	4.31/10 (0–10)

**Table 3:** Overview of the answers on the theory/case mix questions

Q1/ Chain collision, possible cotaminated patients:	
Isolate in distal corner	5 %
In waiting room	49 %
In garage	1 %
**Wait separately outside**	**45 %**
No action, hide	0 %
Q2/ Iodine tablets protect against:	
External radiation	28 %
**Internal radiation**	**15 %**
Both external and internal	24 %
No radiation protection	20 %
Don’t know	13 %
Q3/Tthe CGV means:	
Operational leader of overall disaster management	26 %
Controlling arriving ambulances	4 %
Field hospital supplies	2 %
**Deciding which patients go where**	**14 %**
Don’t know	55 %
Q4/ Postman with necrotic lesions:	
Frostbite	10 %
New chemical product	22 %
**Possible anthrax**	**47 %**
Use of new kind of black ink	1 %
Don’t know	20 %
Q5/ Chemical decontamination:	
Oral antidote	5 %
Antidote body smear	3 %
Antidote spray special miltary cabin	57 %
**Wash with water and soap**	**15 %**
Don’t know	20 %
Q6/ What limits radiation damage the most?	
Protective clothing	3 %
Fast decontamination	1 %
Oral iodine tablets	41 %
**Limit time of exposure, increase distance and shielding**	**54 %**
Don’t know	1 %
Q7/ 2 most important objects to take along in evacuation:	
Smartphone	57 %
Laptop	2 %
**ID/health insurance cards**	**46 %**
Syllabus/handbook	1 %
Sixpack of beer	4 %
**Normally used medication**	**79 %**
Photo of loved one	1 %
None of the above	6 %
Don’t know	0 %
Q8/ Superficial cuts and first degree burns, go to	
Nearest hospital	47 %
Closest hospital with burn unit	5 %
Home (recover and sleep)	6 %
**Hospital ED further away**	**41 %**
Don’t know	1 %
Q9/ First step in nuclear decontamination	
Shower patient	8 %
Administer iodine tablets	53 %
**Take off clothes and shoes**	**23 %**
Put on lead apron	4 %
Don’t know	12 %
Q10/ Traffic accident with 2 trucks and 2 victims, what to do?	
Stop, call 112 and help lying victim	40 %
Stop, call 112 and help limping victim	2 %
**Stop at safe distance and wait for clearance fire brigade**	**54 %**
Drive by and call 112 at hospital	4 %
Do as if nothing happened	0 %

The correct answers are given in bold. The “don’t know” option was added to eliminate wild guess bias

Female and younger students scored better as well as students with prior knowledge or EMS experience. Those expressing the ambition to become a specialist score better than occupational or family physicians. Those who find it absolutely necessary to incorporate Disaster Medicine in the curriculum have a significant lower score than those feeling it useful. There is a very strong correlation between the test score and self-estimated knowledge, self-estimated capability and willingness to respond on the other hand.

There were no significant differences between the faculties, not in demographics nor in scores.

No universities offer any disaster medicine training in their curricula. Some students are informed during internship on EDs with a disaster prone staff but this on a voluntary unstructured base, not linked with the university curricula.

## Discussion

In case of mass casualty incidents, all unaffected, available hands are expected to attend in controlling the situation. So every physician, whatever speciality he or she might have, should be able to assist [17]. When communities get isolated as in natural disasters, the family physicians could be the only source of medical relief until external help is organised [18]. In this option, the Association of American Medical Colleges did recommend that all medical schools should thoroughly educate their students about EMS to ensure coordinated responses to weapons of mass destruction or other public health threats [19]. However, recent evaluation proves that this exposure still is very limited with a call for a national curriculum [20, 21]. Looking at the European situation, there is an established curriculum in Germany [22]. Italy is in the experimental phase testing a programme and educational methods in several medical schools [23] following a clear need expressed by the students [24]. Belgium has a limited introduction in three faculties [25].

Our findings demonstrate that medical students in The Netherlands perform not better compared with their Belgian colleagues. Despite a considerable willingness to respond in case of a disaster, education and training in disaster medicine are inadequate to meet these challenges. The students seem to be aware of this situation as half of the respondents find it absolutely necessary to incorporate it in their regular curriculum. They seem to be most at ease with infectious problems, probably due to the fact that this kind of pathology is discussed in regular lectures on internal medicine or infectiology. Despite media attention after the Fukushima incident, nuclear problems remain the big unknown. Perceived knowledge and capability is limited over different situations, and this was confirmed by the test with practical cases. Misconceptions on (de)contamination and radioprotective effects of iodine tablets create dangerous situations for themselves, patients and other healthcare professionals. Only implementation of a national (or European) curriculum on disaster management, not ready available at time of the study, can solve the problem. Our study however raised the awareness of this problem in one faculty (Rotterdam) where a voluntary basic course is considered.

Comparison with a recent similar survey amongst Belgian senior medical students [25] revealed a lower mean test score, a lower willingness to respond and a lower estimated capability in chemical and infectious incidents in our study population.

Recruiting the students was a major limitation in this project. We could only contact the students by medical faculties with variable levels of cooperation and/or by social media groups. In an era of survey fatigue, this complex procedure will limit participation to really motivated persons so our results may potentially be too optimistic. Anonymous participation in this online survey limits scientific control on participants so eventual duplicate results cannot be excluded. Depending on self-reported information could bias the results as well; however, the strong correlation between estimated knowledge and capability and test score on the other hand limits this assumption. Exclusion of the Amsterdam students could also bias our results. We do hope this effect is limited as there were no differences in demographics and results between all other faculties.

## Conclusions

In conclusion, we could state that Dutch senior medical students do believe in the usefulness of teaching Disaster Medicine in the regular curriculum. Although knowledge and estimated capability are limited, there is a relative high willingness to respond. Development and implementation of European guidelines could help to establish a basic training preparing them for a real incident.

## Additional file

Additional file 1: Figure S1. Survey used to evaluate disaster medicine training/education. (PDF 130 kb)
