# Supplementary material for: Dutch senior medical students and disaster medicine: a national survey
Source: Int J Emerg Med. 2015 Sep 3;8:34. doi: 10.1186/s12245-015-0077-0 (PMC4558995; doi:10.1186/s12245-015-0077-0)
Supplement: Additional file 1: Figure S1. — Survey used to evaluate disaster medicine training/education. (PDF 130 kb) [file 12245_2015_77_MOESM1_ESM.pdf]

Figure 1: Survey used to evaluate disaster medicine training/education.

1/ Gender
- Male
- Female

2/ Age in years

3/ Study year
- 5th
- 6th

4/ What's your university?
- Leiden
- Erasmus Rotterdam
- Groningen
- Radboud Nijmegen
- Maastricht
- Vrije Universiteit Amsterdam
- Amsterdam (AMC)

5/ What's your planned professional orientation?
- Family practice
- Occupational/insurance medicine
- Specialisation
  - Emergency medicine
  - Intensive care
  - Anesthesiology
  - Nuclear medicine
  - Others

6/ Do you live within 20 Kms of a nuclear installation?
- Yes
- No
- Don't Know

7/ Do you live within 20 Kms of a chemical installation?
- Yes
- No
- Don't Know

8/ Do you have any contacts with EMS or Disaster Medicine besides your medical studies?
- Yes
  - First aid group
  - Ambulance service
  - Fire Dept
  - Sigma
  - Army
  - Others
- No

9/ Did you have had any prior education in disaster management?
- Yes
  - University
  - Other sources
  - Both
- No

10/ Do you have any knowledge on how to treat chemical/ nuclear / biological incidents and/or their victims?

Yes

From University courses

From other courses

From internet

From literature

Others

No

11/ Do you think a basic course on disaster medicine should be included in the regular medical curriculum?

Absolutely

Useful

Useless

12/ How (on a scale from 0-10) do you estimate your knowledge on?

Nuclear incidents

Chemical incidents

Biological incidents (eg anthrax)

Epidemiological outbreak of a very contagious disease (eg swine flu)

Epidemiological outbreak of very dangerous and contagious disease (eg Ebola)

13/ How (on a scale from 0-10) do you estimate your capability to deal with?

Nuclear incidents

Chemical incidents

Biological incidents (eg anthrax)

Epidemiological outbreak of a very contagious disease (eg swine flu)

Epidemiological outbreak of very dangerous and contagious disease (eg Ebola)

14/ If, during your appenticeship, you should be confronted with one of following scenarios would you participate (on a scale from 0-10) in the care for these patients?

Nuclear incidents

Chemical incidents

Biological incidents (eg anthrax)

Epidemiological outbreak of a very contagious disease (eg swine flu)

Epidemiological outbreak of very dangerous and contagious disease (eg Ebola)

15/ During your appenticeship a mass casualty incident from a chain collision between cars, trucks and a bus happened near your hospital. You are summoned to triage the victims at the entrance of the emergency department. 4 persons with minimal or no injuries in wet clothings with an irritating smell come walking by. What would you do?

- You put them in the far edge of the ED so the odor does not disturb other patients
- You put them in the waiting room and concentrate yourself on the seriously wounded patients
- You let them wait in the garage behind the triage so the smell is limited to that space.
- You put them aside before the entrance and fetch a supervisor
- You don't do anything and go hide yourself somewhere in the hospital

16/The government provides free iodine tablets to be used in case of a nuclear incident. Do you know to what they will protect you?

- Against external radiation
- Against internal radiation
- Against internal and external radiation
- They don't protect against radiation at all
- I don't know.

17/ Your supervisor takes you with hem on prehospital intervention for a mass casualty incident. Once on the terrain he sends you to assist the "CGV" while he starts the triage. Do you know who you're going to help?

- You assist the operational coordinator of the disaster relief
- You assist the person who directs all incoming ambulances
- You assist the responsable for the supplies of your medical post
- You assist the person who decides which victim is directed to which hospital wihth available transportation means
- I don't know at all.

18/At the ED of the 3rd hospital you work you see (again as in the 2 former hospitals) a postman with limited black, necrotic skin lesions at his hands. What do you think?

- Frostbite as indeed, it's a very cold winter
- Use of a new and agressive chemical in post handling
- Possible malicious spread of anthrax through mail, i fetch my supervisor
- Use of a new kind of black ink
- I don't know at all.

19/ How to decontaminate a patient after a chemical incident?

- Peroral antidote
- Body smear with specific antidote
- Specific total body antidotespray in a special military cabin
- Wash/shower with water and soap
- I don't know.

20/ How to limit radiation damage the best/most?

- Use of personal protective equipment
- Quick decontamination
- Iodine tablets orally
- Limiting exposure time, shielding and maximalising distance to the source.
- I don't know

21/ Due to a flooding you and you fellow students have to be evacuated from your student hostel. What are the 2 most important things to take with you (for everyone, not only personal!!)?

- Smartphone
- Laptop
- ID card or medical insurance card
- Courses, handbook
- Sixpack beer
- Routinely used medication or a list of it
- A picture of your loved one
- None of these
- I don't know

22/ At the latest student party there has been an explosion in the party hall with multiple victims (even deaths and seriously injured victims). You were lucky being at the exit at the moment of the blast and you're not injured at all. 3 of your friends have some superficial cuts from the glass and first degree burns at their forearms. You can drive them with your own car, where will you go?

- The nearby hospital so everybody stays together
- The nearest burn centre
- To your rooms to recover from the emotions
- An emergency department further away
- I don't know.

23/ What’s the very first step in decontaminating a patient from a nuclear incident?

- Showering
- iodine tablets orally
- Removal of clothing and shoes
- Giving the patient a lead apron to wear
- I don’t know.

24/On your way to the hospital you cross an accident scene with 2 trucks. There is a jet of fluid from the fluid cannister of the first truck, a tanker. A patient is lying on the road next to this truck with some movements. The driver of the other truck is limping around trying to get your attention. What would you do?

- Stop, call 112 and go to check the patient on the ground.
- Stop, call 112 and go to check the limping patient, after that i will check te one on the ground.
- Stop at a safe distance and wait untill the firemen clear the site before checking the patients
- Continue my way and call 112 as soon as i’m at the hospital
- Continue my way as if nothing happened.
